# Scraps

**Published:** 1900-06

**Authors:** 


### SCRAPS

PICKED UP

BY L. M. G.

---

**Clinical Records** (31).—Doctor: "Are you feeling very ill? May I see your tongue?" Patient: "That's no use, doctor; no tongue can tell how bad I feel."

**Medical Philology** (XXXIV.).—"Dote," as a verb, is now almost exclusively used to express excessive fondness, but in its earlier use it was applied to the condition now referred to in the familiar terms "dotage" or "dotard."

The *Catholicon* has, "to dote; *desipere, desipiscere.*" Mr. Herrtage in his note quotes:—

"To dote, *delirare.*" Manip. Vocab.

"Me þuncheȝ þe alde mon wole dotie." Laȝamon, i. 140.

"His mouth slavers, his tethe rotes, His wyttes fayles, and he ofte dotes."

Pricke of Conscience, l. 785.

"*Radoter.* To dote, rave, play the cokes, erre grossly in vnderstanding." Cotgrave.

C. L. G., writing in *The Londoner* of May 5th, says that "Mr. Treves's attack on the society ladies at the front, who with the flies constituted the two great plagues of South Africa, must be my excuse for the following:—

There was a young *belle* from North Berwick,
Whose conduct was highly hysteric;
She followed the guns,
And distributed buns
To the men who were down with enteric.

"Sir Walter's famous eulogy will, we fear, have to be revised in accordance with the condemnation of Mr. Treves—which, by the way, has already elicited a penetrating shriek of protest from the *Daily Female.* Henceforth it must run:—

O woman! in our hours of ease
Uncertain, coy, and hard to please;
When pain and anguish wring the brow,
More terrible than flies art thou."

**Dr. Weir Mitchell as a Poet.**—The Congress of American Physicians and Surgeons, held on May 3rd, produced some interesting communications. Dr. S. Weir Mitchell, whose literary accomplishments are well known, wrote for it and read a poem entitled "The Physician," the whole of which is printed in the *Philadelphia Medical Journal* of May 5th. After a historic glance at the sources of Medicine, we read—

"Too oft our changeful story seems to show
That what men knew they only seemed to know.
They lived, they toiled, they joined the silent dead.
On dusty shelves their books repose, unread.
The scholar wandering o'er this vast domain
Once rich with living thought, may think how vain
Our work will seem to those who hither come
To sum our gains when we, in turn, are dumb.
Yet that which wins to-morrow's grateful praise
Is the sure child of faltering yesterdays,
And countless hands must till the stubborn soil
That one may reap the harvest of their toil."

Jenner and Harvey receive appreciative notice, and we are urged to follow their steps:—

"Know the true children of the mighty dead
Are they alone who in their footsteps tread,
And that a man's true ancestors are they
Who, dying, left him all that genius may.
You wield new arms, are 'neath new flags arrayed,
Yet you are still what these our fathers made."

We are reminded that—

"God's ways are dark, and in their gloom we walk;
Not ours to know why life's grim spectres stalk.
We tread mysterious paths in touch with pain,
Birth, death, disease, strange phantoms of the brain.
Perplexed we recognise the doubtful hour
When indecision paralyses power.

. . .

Our ancient lesson will be ever new;
That priceless lesson will be ever true;
Time did not teach it: time will change it not,
This, this shall last, though all our love's forgot,
To give what none can measure, none can weigh,
Simply to go where duty points the way;
To face unquestioning the fever's breath,
The hundred shadows of the vale of death;
To bear Christ's message through the battle's rage,
The yellow plague, the leper's island cage,
And with our noblest 'well to understand
The poor man's call as only God's command.'"

Dr. Mitchell lamented that those to whom we are most indebted do not always receive their meed of praise, and he pays his tribute to a pioneer of anæsthesia:

"We are but mortal, and, with blinded eyes,
May fail to see who surely won the prize,
Or see too late, as once we saw in vain
The fate of him who wrought the death of pain.
Guard well that memory, lest again we flout
Some hero-victim with our torturing doubt.
How thanked we Morton? Ah! 'No joy-bells rang,
No pæons greeted, and no poet sang.
No cannon thundered from a peaceful strand
That bloodless victory to a grateful land.
We took the gift, so humbly, simple given,
And, coldly doubting, left the rest to Heaven.'"

Dr. Weir Mitchell also wrote a poem addressed to Dr. Abraham Jacobi, whose seventieth birthday has just been celebrated at a banquet in New York attended by four hundred of his friends and professional associates. During the evening there was presented to Dr. Jacobi a "Festschrift" entitled *International Contributions to Medical Literature*, which contains a large number of original papers by eminent physicians and scientists in all parts of Europe and America. More than a year was taken up in the preparation of the volume, which is believed to be the first of its kind published in America. The Bristol Medical Library has received a presentation copy. I quote four stanzas from Dr. Mitchell's poem, which is printed in full in the *Boston Medical and Surgical Journal* of May 10th and in the *Medical News* of May 12th:

"*Ave Magister!* Take from us to-night
The well-earned praise of all who love our art
For this long lesson of unending work,
For strength of brain and precious wealth of heart.

"Your busy hand gives much; but, oh, far more,
The gallant soul that teaches how to meet
Unfriended exile, sorrow, want and all
That crush the weak with failure and defeat.

"*Ave Amicus!* If around this board
Are they who watched you thro' laborious years,
Beyond these walls, in many a grateful home,
Your step dismissed a thousand pallid fears.

"That kindly face, that gravely tender look,
Thro' darkened hours how many a mother knew!
And in that look won sweet reprieve of hope,
Sure that all earth could give was there with you."

